# Targeting Lipocalin-2 in Inflammatory Breast Cancer Cells with Small Interference RNA and Small Molecule Inhibitors

**DOI:** 10.3390/ijms22168581

**Published:** 2021-08-10

**Authors:** Ginette S. Santiago-Sánchez, Ricardo Noriega-Rivera, Eliud Hernández-O’Farrill, Fatma Valiyeva, Blanca Quiñones-Diaz, Emilly S. Villodre, Bisrat G. Debeb, Andrea Rosado-Albacarys, Pablo E. Vivas-Mejía

**Affiliations:** 1 Department of Biochemistry, Medical Sciences Campus, University of Puerto Rico, San Juan 00936, Puerto Rico; ginette.santiago@upr.edu (G.S.S.-S.); ricardo.noriega1@upr.edu (R.N.-R.); blanca.quinones@upr.edu (B.Q.-D.); 2 Comprehensive Cancer Center, Medical Sciences Campus, University of Puerto Rico, San Juan 00936, Puerto Rico; fvaliyeva@cccupr.org; 3 Department of Pharmaceutical Sciences, Medical Sciences Campus, University of Puerto Rico, San Juan 00936, Puerto Rico; eliud.hernandez@upr.edu; 4 Department of Breast Medical Oncology, The University of Texas, MD Anderson Cancer Center, Houston, TX 77030, USA; esschlee@mdanderson.org (E.S.V.); bgdebeb@mdanderson.org (B.G.D.); 5 Department of General Sciences, Rio Piedras Campus, University of Puerto Rico, San Juan 00936, Puerto Rico; andrea.rosado2@upr.edu

**Keywords:** lipocalin-2, LCN2, IBC, inflammatory breast cancer, siRNA, small molecule inhibitors, docking

## Abstract

Inflammatory Breast Cancer (IBC) is an aggressive form of invasive breast cancer, highly metastatic, representing 2–4% of all breast cancer cases in the United States. Despite its rare nature, IBC is responsible for 7–10% of all breast cancer deaths, with a 5-year survival rate of 40%. Thus, targeted and effective therapies against IBC are needed. Here, we proposed Lipocalin-2 (LCN2)—a secreted glycoprotein aberrantly abundant in different cancers—as a plausible target for IBC. In immunoblotting, we observed higher LCN2 protein levels in IBC cells than non-IBC cells, where the LCN2 levels were almost undetectable. We assessed the biological effects of targeting LCN2 in IBC cells with small interference RNAs (siRNAs) and small molecule inhibitors. siRNA-mediated LCN2 silencing in IBC cells significantly reduced cell proliferation, viability, migration, and invasion. Furthermore, LCN2 silencing promoted apoptosis and arrested the cell cycle progression in the G0/G1 to S phase transition. We used in silico analysis with a library of 25,000 compounds to identify potential LCN2 inhibitors, and four out of sixteen selected compounds significantly decreased cell proliferation, cell viability, and the AKT phosphorylation levels in SUM149 cells. Moreover, ectopically expressing LCN2 MCF7 cells, treated with two potential LCN2 inhibitors (ZINC00784494 and ZINC00640089) showed a significant decrease in cell proliferation. Our findings suggest LCN2 as a promising target for IBC treatment using siRNA and small molecule inhibitors.

## 1. Introduction

Inflammatory Breast Cancer (IBC) is an aggressive locally advanced breast cancer (LABC) subtype that disproportionately affects younger women and has a poor survival outcome [1,2,3]. IBC comprises approximately 2–4% of all breast cancer cases in the United States and accounts for 7–10% of all breast cancer-related deaths [2,3]. The aggressive nature of IBC has been attributed to the high risk of both distant metastasis and locoregional (lymph node and skin) recurrence [4,5]. Moreover, all molecular subtypes of IBC are more aggressive compared to non-IBC subtypes, having low recurrence-free survival, low overall survival (40% versus 65% for non-IBC patients), and poor therapeutic response [3,5,6,7,8].

Current IBC treatment includes a multimodal approach consisting of neoadjuvant chemotherapy (NAT), followed by surgery and postmastectomy radiation therapy [3,9]. The NAT-therapeutic strategy is based on IBC molecular profiles, including anthracycline-based and/or taxane-based therapy [3,10]. The incidence of human epidermal growth factor receptor 2 (HER2) and triple-negative breast cancer (TNBC) subtypes are high in IBC [2,11,12]. In the case of HER2-positive subtypes, additional HER2-targeted therapy is used [10]. However, the main limitation facing IBC treatment is the lack of specific therapeutic targets.

Lipocalin-2 (LCN2) is a secreted glycoprotein involved in transporting hydrophobic ligands across the cell membrane, modulating the immune response during bacterial infection, and promoting epithelial cell differentiation and iron homeostasis [13]. LCN2 is aberrantly upregulated in cancerous tissues derived from the pancreas, colon, ovaries, and breast [13,14,15,16,17,18]. Overexpression of LCN2 is also associated with the progression of aggressive forms of endometrial carcinoma, pancreas, and breast cancers [17,19,20]. Particularly, LCN2 is aberrantly abundant in inflammatory breast cancer (IBC) patients independent of molecular subtype differences [21]. However, the biological consequences of targeting LCN2 using siRNAs or small molecule inhibitors in IBC have not been studied.

In this study, we compared the expression of LCN2 in IBC and non-IBC cells and explored the potential benefits of targeting LCN2 in IBC. LCN2-siRNA-based silencing in IBC cells induced apoptosis and cell cycle progression arrest and decreased colony formation, migration, and invasion. In addition, we used a structure-based virtual screening approach to identify potential chemical inhibitors of LCN2. We used a set of 25,000 ligands from the Asinex library and identified 16 potential LNC2 inhibitors. Molecular docking achieved interactions between residues within the three pockets of the LCN2-calyx and the ligands. We observed that two compounds (ZINC00784494 and ZINC00640089) reduced cell viability and colony formation of IBC cells. Further specificity of the inhibitors was observed when MCF7 cells (non-IBC cells) were ectopically transfected with LNC2 and incubated with the two inhibitors. These findings suggest LCN2 as a potential therapeutic target against IBC.

## 2. Results

### 2.1. LCN2 Protein Levels Are Increased in Inflammatory Breast Cancer Cell (IBC) Lines

**Figure 1:**
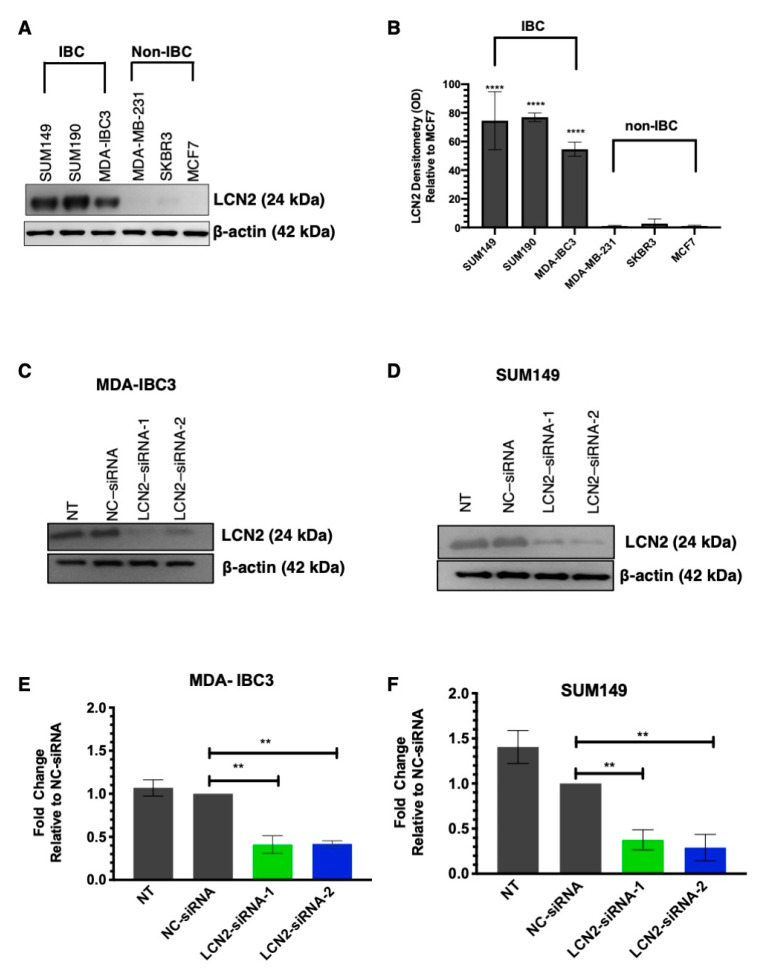
siRNA-based silencing in LCN2-overexpressing IBC cells. (**A**) Western blot analysis for LCN2 and β-actin (as loading control) in a panel of IBC (SUM149, SUM190, and MDA-IBC3) and non-IBC (MDA-MB-231, SKBR3, and MCF7) cells. (**B**) Densitometric analysis of band intensities was performed, and values were calculated relative to non-IBC cells, MCF7. Results are shown as Mean ± SEM of triplicate experiments, **** *p* < 0.001). Two different siRNAs targeting exon 3 and exon 5 of the human LCN2 sequence (NC_000009.12) were used. Western blot analysis of (**C**) MDA-IBC3 cells and (**D**) SUM149 cells were performed after transiently transfected with LCN2-siRNA-1, LCN2-siRNA-2, and negative control-siRNA (NC-siRNA) at 100 nmol/L concentration, as described in materials and methods. Non-treated (NT) cells were treated with the transfection reagent. Densitometric analysis of band intensities of (**E**) MDA-IBC3 and (**F**) SUM149 cells was calculated relative to the NC-siRNA. Results are shown as Mean ± SEM of triplicate experiments (** *p* < 0.01).

First, we measured the LCN2 protein levels in a panel of IBC and non-IBC cell lines. The description of the cell lines, including ER, PR, and HER-2 status, are shown in Table S1. Western blot analysis showed significantly higher LCN2 protein levels in IBC cells than non-IBC cells (Figure 1A). Densitometric analysis of band intensities confirmed our findings (****, *p* < 0.0001, Figure 1B).

### 2.2. LCN2-siRNA-Based Silencing Reduces Cell Colony Formation, Cell Invasion and Migration in IBC Cells

We determined the biological effects of siRNA-mediated LCN2 silencing in IBC cells, MDA-IBC3 (HER2+) and SUM149 (TNBC) IBC cells. Western blot analysis of MDA-IBC3 showed a significant decrease in LCN2 protein levels in cells transiently transfected with siRNAs compared to controls (Figure 1C). Densitometric analysis of band intensities showed a decrease of LCN2 protein levels of 59% and 58% with LCN2-siRNA-1 and LCN2-siRNA-2, respectively (Figure 1D, ** *p* < 0.01). Similar results were obtained when LCN2 was silenced in SUM149 cells (Figure 1E). Densitometric analysis showed a decrease of LCN2 protein levels of 62% and 71% with LCN2-siRNA-1 and LCN2-siRNA-2, respectively (** *p* < 0.01, Figure 1F).

**Figure 2:**
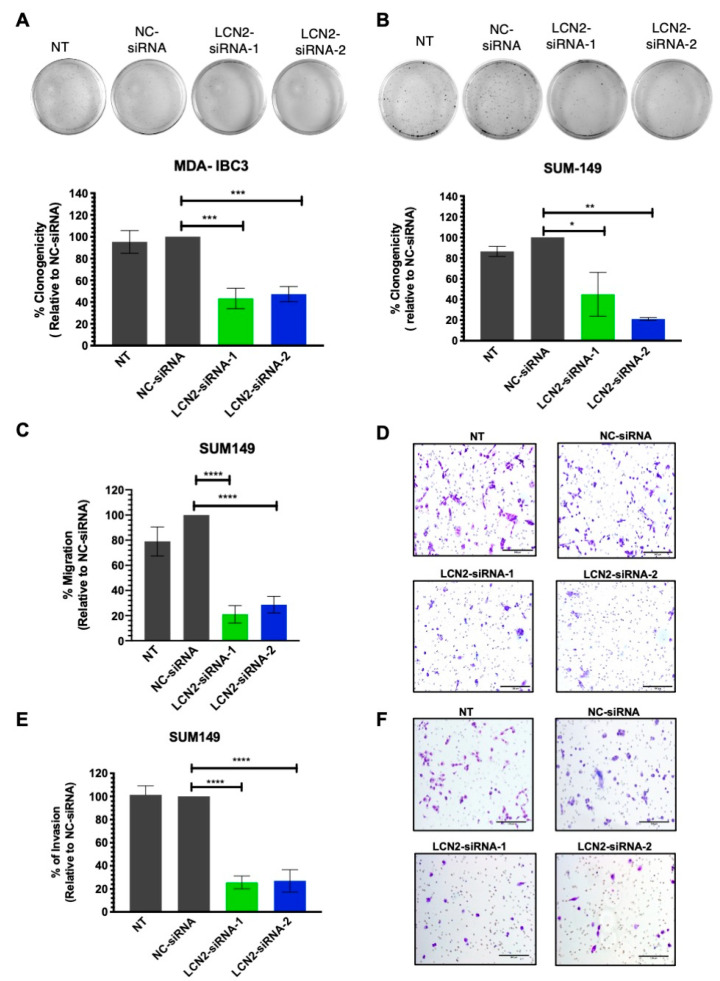
LCN2-siRNA-based silencing inhibits colony formation, migration, and invasion of IBC cells. Colony formation assay was performed after LCN2-siRNA-based silencing in MDA-IBC3 and SUM149 cells. Cell proliferation was performed in (**A**) MDA-IBC3 cells and (**B**) SUM149 cells. Results are shown as Mean ± SEM of triplicate experiments (* *p* < 0.05, ** *p* < 0.01, *** *p* < 0.001). (**C**) Migration assay was performed after LCN2-siRNA transfection (100 nM siRNA, final concentration) in SUM149. (**D**) NC-siRNA cells represent 100% migration. Images of migrated cells were taken at 20× magnification, scale bar = 100 µm. Results are shown as Mean ± SEM of triplicate experiments (**** *p* < 0.0001). (**E**) Invasion assay was performed after LCN2-siRNA transfection (100 nM siRNA, final concentration) in SUM149 cells. (**F**) NC-siRNA cells represent 100% invasion. Images of invaded cells were acquired with a light microscope 20× magnification, Scale bar = 100 µm. Results are shown as Mean ± SEM of triplicate experiments (**** *p* < 0.0001).

To assess the long-term effect of LCN2 silencing in IBC cells, we performed colony formation assays. Transient transfection of LCN2-siRNAs on MDA-IBC3 cells significantly reduced the number of colonies compared with the negative control siRNA (NC-siRNA) (LCN2-siRNA-1: 57%; LCN2-siRNA-2: 53% reductions on colony formation, ** *p* < 0.01, Figure 2A). LCN2 silencing in SUM149 significantly decreased the number of colonies compared with the NC-siRNA (LCN2-siRNA-1: 45% and LCN2-siRNA-2: 79% reductions on colony formation, * *p* < 0.05, ** *p* < 0.01, respectively; Figure 2B).

As overexpression of LCN2 has been associated with increased metastasis of cancer cells [17,22,23], we assessed the effect of LCN2 silencing in the migration and invasiveness potential of IBC cells. A significant reduction in the migration of SUM149 cells was observed following LCN2 silencing compared to NC-siRNA (LCN2-siRNA-1: 79% and LCN2-siRNA-2: 71% reductions; **** *p* < 0.0001, Figure 2C,D). In the transwell invasion assays, we observed a significant reduction in the number of invaded cells compared to NC-siRNA (LCN2-siRNA-1:77% and LCN2-siRNA2: 71% reductions, **** *p* < 0.0001, Figure 2E,F). We did not observe visible changes in the short-term viability of MDA-IBC3 or SUM149 cells after LCN2 silencing (Figure S1A,B). This evidence suggests that LCN2-siRNA silencing has more pronounced effects on cell growth, proliferation, and invasion than in the cell viability of IBC cells.

### 2.3. LCN2 Silencing Induces Apoptosis and Cell Cycle Arrest

**Figure 3:**
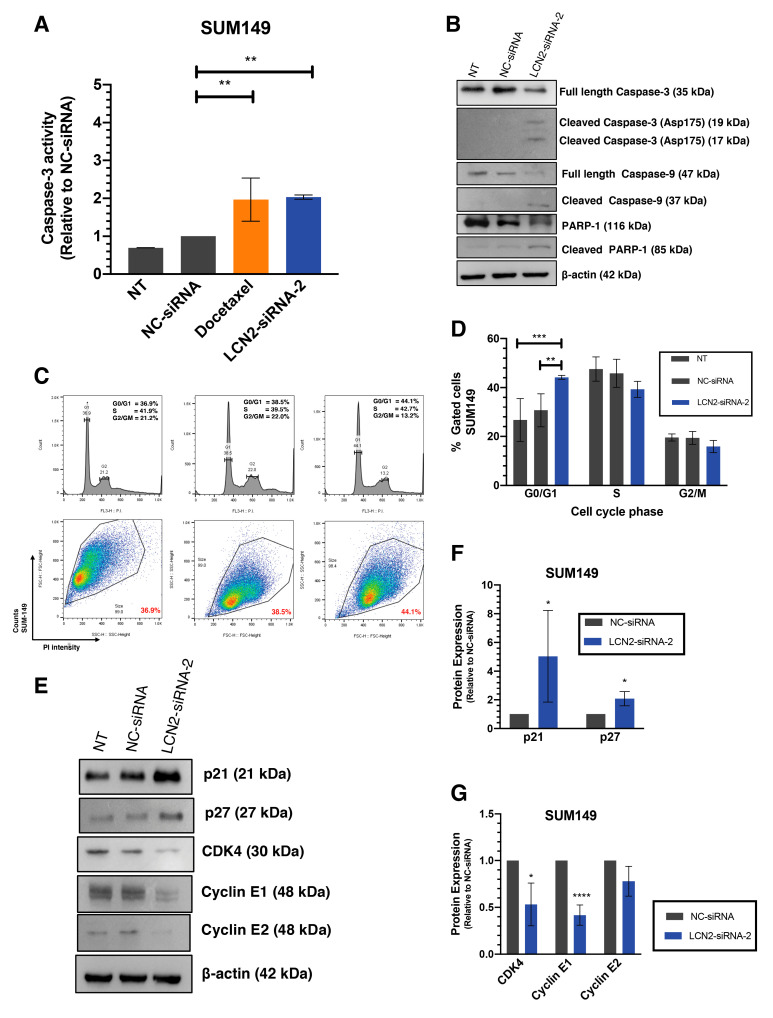
LCN2-siRNA-based silencing induces apoptosis and cell cycle arrest in IBC cells. SUM149 cells were transfected with 100 mM of negative control (NC-siRNA) or LCN2 siRNA (siRNA-2). (**A**) Caspase-3 fluorometric activity assay in SUM149 cells 72 h after LCN2-siRNA-2 and NC-siRNA transfection. Docetaxel (0.5 nM final concentration) was used as a positive control. (**B**) Western blot analysis of apoptotic-related proteins. (**C**) Histogram showing cell cycle arrest at G0/G1 to S phase transition after LCN2-siRNA-2 transfection in SUM149 cells compared with NC-siRNA. (**D**) Quantification of the flow cytometry data showed an increase in SUM149-LCN2-siRNA-2 transfected cells at G0/G1 to S phase transition. (**E**) Western blot analysis of cell cycle-related proteins 72 h after siRNAs transfection. (**F**,**G**) Densitometric analysis of the band intensities showed in E. Results are shown as Mean ± SEM of triplicate experiments (* *p* < 0.05, ** *p* < 0.01, *** *p* < 0.001, **** *p* < 0.0001).

We next investigated whether the reduction in colony formation after LCN2 silencing was due to the activation of apoptosis, cell cycle arrest, or both. We measured the activation of caspase-3 as the indicator of apoptosis. Compared with NC-siRNA, silencing of LCN2 with siRNAs in SUM149 cells resulted in a 2-fold increase in Caspase-3 activity (** *p* < 0.01, Figure 3A). Similar results were observed after LCN2 silencing in MDA-IBC3 cells, where around a 3-fold increase in caspase-3 activity was observed (Figure S2). Docetaxel, a common drug used for IBC treatment, used here as a positive control, also resulted in a two-fold increase of caspase-3 activity [24].

Activation of apoptosis was confirmed by measuring changes in apoptotic-related proteins by western blot analysis. Transient transfection of LCN2-siRNA-2 achieved a reduction of full-length Caspase-3 and full-length Caspase-9, and a significant increase in the active form of Caspase-9 (cleaved Caspase-9) and Caspase-3 (cleaved Caspase-3). Moreover, a reduction in the poly-ADP ribose polymerase-1 (PARP-1) full-length band intensity, together with an increase in the cleaved PARP-1 band compared with NC-siRNA, was observed (Figure 3B).

Then, we assessed cell cycle progression after LCN2 silencing in SUM149 cells by flow cytometry. Cell cycle arrest in the G0/G1 to S phase was observed in SUM149 cells, 72 h post-transfection (** *p* < 0.01, *** *p* < 0.001, Figure 3C,D). These results were confirmed by studying the changes of key proteins involved in the G0/G1 to S phase transition by western blot analysis. Notably, an increase in the protein levels of tumor suppressors p21 and p27 was observed (* *p* < 0.05, Figure 3E,F). Moreover, a reduction of the checkpoint proteins of S phase –cyclin E1, cyclin E2, and CDK4– was observed (* *p* < 0.05, **** *p* < 0.0001, Figure 3E,G). Compared with NC-siRNA, transient transfection of LCN2-siRNA-2 in SUM149 cells showed a tendency in cell cycle arrest in G0/G1 to S phase, 48 h post-transfection (Figure S3A,B).

### 2.4. Identification of LCN2 Small Molecule Inhibitors by In-Silico Analysis

**Figure 4:**
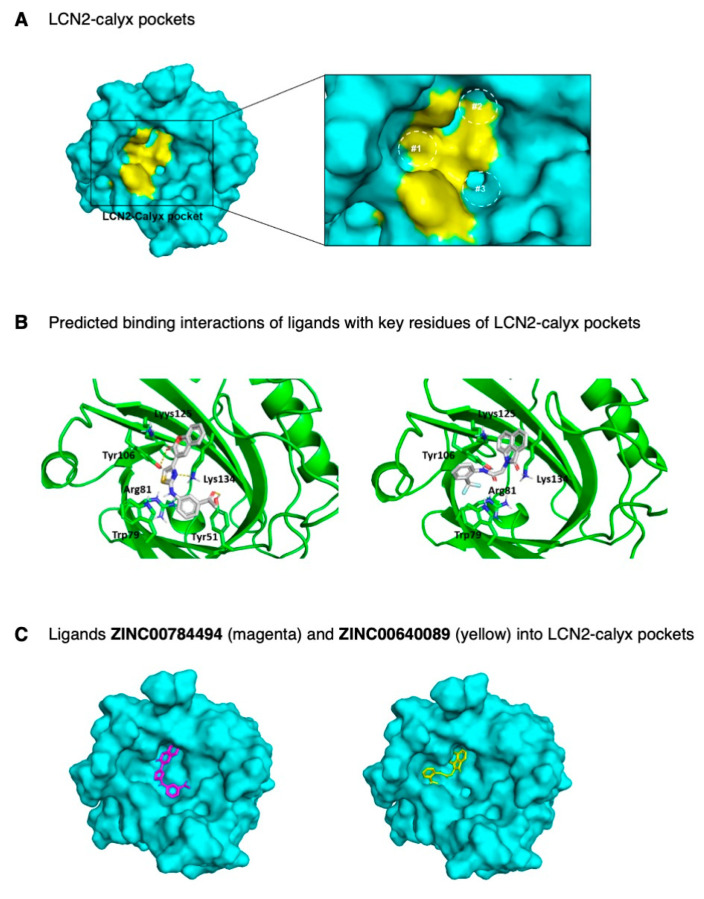
Molecular model and docking of ZINC00784494 and ZINC00640089 ligands into LCN2-calyx pocket. (**A**) Surface model representation of LCN2-calyx pockets. Pockets #1, #2, and #3 (dotted circles) are represented with key amino acid residues in yellow color (right panel). (**B**) Cartoon docking representation and predicted binding interactions of ligands with key residues of LCN2-calyx pocket. Interactions are represented with yellow dotted lines. Residues are displayed with a three-letter code and numbers representing the position in the polypeptide. (**C**) Surface docking representation of ligands (represented as sticks) ZINC00784494 (magenta) and ZINC00640089 (yellow) into the LCN2-calyx pocket.

To identify lead compounds that potentially target LCN2, we analyzed the structural properties of the crystal structure of the LCN2-calyx pocket and ligand-bound structures (28). The LCN2-calyx comprises three pockets (Pockets #1, #2, and #3 of Figure 4A) that accommodate critical functional groups for siderophores, which creates specificity for ligand recognition [25,26]. The key siderophore-contacting residues are Trp79, Arg81, Tyr106, Lys125, and Lys134. Moreover, the side chains of residues Trp79 and Arg81 increase flexibility at the LCN2-calyx allowing the accommodation of different ligands in the protein pocket [25,26].

Structure-based virtual screening and molecular docking studies were carried out between the LCN2 protein and a set of 25,000 ligands from the Asinex library using the PyRx virtual screening tool [27]. After docking these compounds into LCN2, the results display various modes of ligand-receptor interactions generated with a docking score. With a binding energy cut-off of −9.6 kcal/mol, a total of 265 hits (1.1% of total ligands) were identified, with the least binding energy ranging from −11.5 to −9.6 kcal/mol. The ligands with a binding energy of −9.6 kcal/mol or less were visualized using the PyMol molecular graphics system [28]. Next, the selected 265 ligands were submitted to the Swiss-ADME server [29], and the list of the best candidates was refined according to the drug-likeness score using the Lipinski rule of five, physicochemical properties, lipophilicity, water-solubility, pharmacokinetics, and the pan-assay interference (PAINS) filter for the identification of potentially problematic fragments (Table S2). The structure-based screening resulted in the selection of 138 molecules with a binding energy range between −11.5 to −10.0 kcal/mol. The 138 ligands were re-ranked based on structural characteristics, predicted binding geometries (docking poses) using PyMOL, and on the main interactions between key residues at the binding site of the LCN2-calyx pocket and the selected ligands. The presence of polar interactions of ligands with Trp79, Arg81, Tyr106, Lys125, and Lys134 was used as a selection criterion, in addition to other polar interactions and stereochemical complementarity. Finally, a total of 25 ligands were selected with a binding affinity ranging between −11.5 to −10.3 kcal/mol, from which 16 ligands were commercially available and used for further in vitro analysis (Table S3). Results of the docked complexes indicated that the binding sites of these ligands interact between side chains of residues of the LCN2-calyx pocket (Figure 4B,C). According to Figure 4B, the predicted binding of ligand ZINC00784494 with LCN2-calyx (−10.4 kcal/mol) was through hydrogen-bonding of the thiazole ring with the side chain -NH of Lys134, and the carbonyl group of the chromenone moiety with the phenol group of Tyr106 residue, and hydrophobic π-interactions of the phenyl ring with Trp79 indole group (Figure 4B).

Similarly, compound ZINC00640089 binds to LCN2-calyx (−10.6 kcal/mol) by positioning the 2-oxo-benzoindole ring near Lys134 for possible hydrogen bonding between the carbonyl group and the -NH group. The carbonyl group of the acetamide moiety of ligand ZINC00640089 is also in proximity for favorable hydrogen bonding with the phenolic group of Tyr106 (Figure 4B). As seen from the docking representations as surface models (Figure 4C), both compounds occupy two pockets into the LCN2-calyx binding site, predicting the potential to block the interaction between LCN2 with its natural ligands.

### 2.5. LCN2-Inhibitors Reduce Colony Formation and Cell Viability in SUM149 Cells

**Figure 5:**
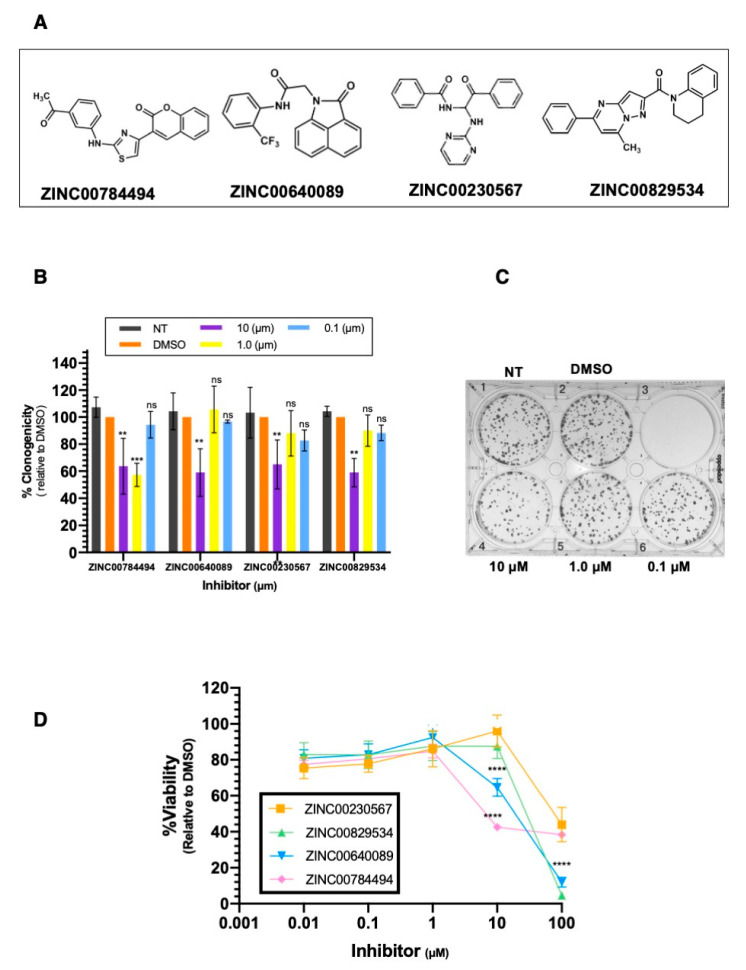
LCN2 inhibitors reduce cell proliferation and cell viability in IBC cells. (**A**) For colony formation assays SUM149 cells were treated with LCN2 inhibitors at 10 µM, 1 µM, and 0.1 µM concentration. (**B**) The percentage of clonogenicity was calculated relative to DMSO. Results are shown as Mean ± SEM of triplicate experiments (** *p* < 0.01, *** *p* < 0.001). (**C**) Representative plate showing a colony formation assay of SUM149 cells treated with the LCN2 inhibitor. (**D**) Cell viability was assessed in SUM149 cells with Alamar Blue dye 72 h after LCN2 inhibitor treatment. The percentage of cell viability was calculated relative to DMSO. Results are shown as Mean ± SEM of triplicate experiments (**** *p* < 0.0001).

Clonogenic assays were used to investigate the effect of the selected compounds on the self-renewing capacity of SUM149 cells. Sixteen compounds were selected through structure-based screening (Table S3). SUM149 cells were seeded and 24 h later they were treated with each inhibitor at different concentrations (10 µM, 1 µM, and 0.1 µM). Four out of the 16 compounds significantly decreased the number of colonies formed (Figure 5A). Particularly, the compound ZINC00784494 showed a significant decrease in the number of colonies formed at 10 µM (37% reduction), and 1 µM (43% reduction) compared to Dimethyl sulfoxide (DMSO) (0.20%, final concentration) (** *p* < 0.01, *** *p* < 0.001, Figure 5B). Compounds ZINC00784494, ZINC00640089, ZINC00230567, and ZINC00829534 significantly reduced the number of colonies formed at 10 µM (42%, 62%, and 41% reduction, respectively) compared to DMSO (** *p* < 0.01, *** *p* < 0.001, Figure 5B). Any of the four compounds significantly reduced the number of colonies at concentrations of 1 µM or 0.1 µM (Figure 5B). Figure 5C is a representative plate showing the changes observed in colony formation with the compounds ZINC00784494, ZINC00640089, ZINC00230567, and ZINC00829534. The additional 12 compounds tested did not show changes in cell proliferation at any of the tested concentrations (Figure S4).

We further assessed the cell viability of SUM149 cells with the four compounds that significantly reduced the number of colonies (Figure 5D). As compared with DMSO, the four compounds significantly reduced cell viability at 100 µM (60% reduction, **** *p* < 0.0001, Figure 5D). Inhibitors ZINC00784494 and ZINC00640089 significantly reduced cell viability at 10 µM. (57%, 35% reduction, respectively, **** *p* < 0.0001, Figure 5D). Any of the four compounds reduced cell viability at concentrations of 1 µM or lower (Figure 5D).

### 2.6. The LCN2 Inhibitors ZINC00784494 and ZINC00640089 Reduced the p-Akt Levels in SUM149 Cells

**Figure 6:**
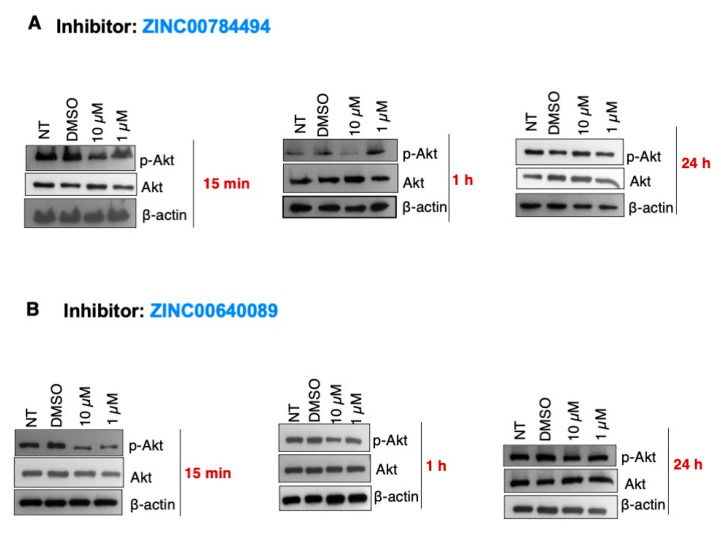
LCN2 inhibitors ZINC00784494 and ZINC00640089 reduced p-Akt in a dose-dependent manner in SUM149 cells. SUM149 cells were incubated with each inhibitor as described in the “materials and methods” section. Changes in AKT and p-AKT protein levels were measured by Western blot with specific antibodies against these protein forms. (**A**) ZINC00784494, (**B**) ZINC00640089.

Evidence indicates that LCN2 activates the EGFR/AKT, a critical pathway regulating the growth, survival, proliferation, and differentiation of mammalian cells [4,5]. Thus, we assessed the effect of LCN2 inhibitors on the phosphorylation levels of AKT. SUM149 cells were treated with the LCN2 inhibitors ZINC00784494 and ZINC00640089 at 10 µM and 1 µM. NT cells and cells treated with DMSO (0.2% final concentration) were used as controls. As compared to DMSO, 10 µM and 1 µM of the LCN2 inhibitor ZINC00784494 reduced the p-Akt protein levels 15 min and 1 h after drug treatment (Figure 6A). Changes in the p-Akt protein levels were not observed 24 h after drug treatment (Figure 6A). Similarly, a reduction in p-AKT protein levels was observed at 10 µM and 1 µM concentrations with the LCN2 inhibitor ZINC00640089. The total Akt protein levels were unaltered in all of the doses and time-points tested (Figure 6A,B).

### 2.7. LCN2-Inhibitors ZINC00784494 and ZINC00640089 Showed Specificity toward LCN2

**Figure 7:**
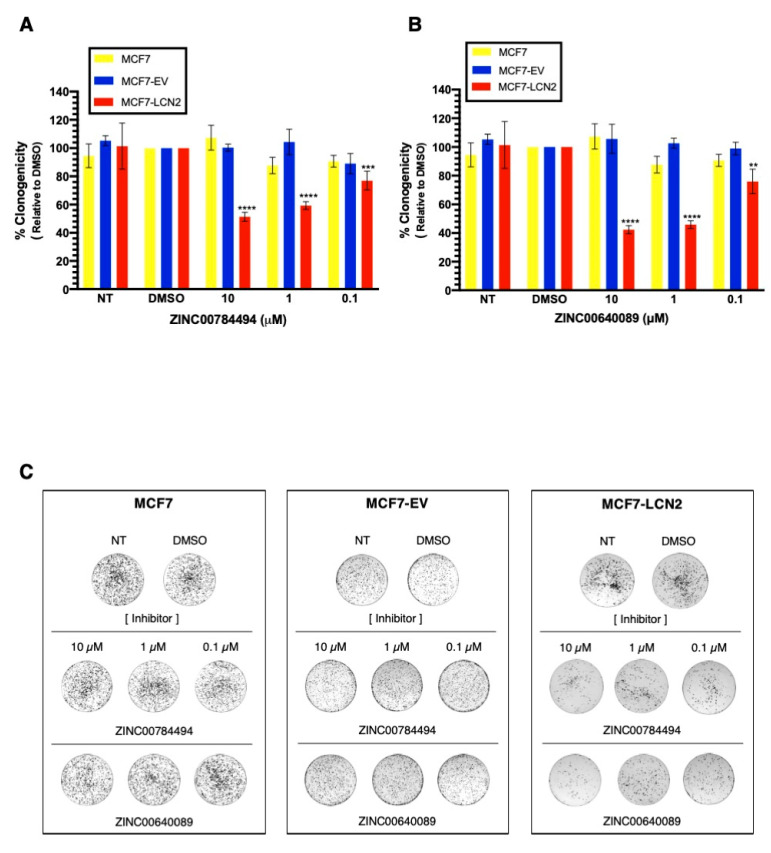
LCN2-inhibitors ZINC00784494 and ZINC00640089 showed specificity toward LCN2-calyx. Cell proliferation in MCF7, MCF7-EV and, MCF7-LCN2 cells after treatment with (**A**) ZINC00784494 inhibitor and (**B**) ZINC00640089 inhibitor. (**C**) A representative clonogenic assay of MCF7, MCF7-EV, and MCF7-LCN2 treated with ZINC00784494 and ZINC00640089. Results are shown as Mean ± SEM of triplicate experiments (** *p* < 0.01, *** *p* < 0.001, **** *p* < 0.0001).

To further study the selectivity of the compounds towards LCN2, MCF7 ectopically expressing LCN2 were exposed to ZINC00784494 and ZINC00640089 inhibitors. Figure S5 shows the LCN2 expression in MCF7, MCF7-EV, and MCF7-LCN2 cells. For comparison purposes, all cells were treated with DMSO at 0.2%. We observed a significant reduction in colony formation of MCF7-LCN2 cells treated with ZINC00784494 at 0.1 μM, 1 μM, and 10 μM concentration compared with untreated cells (23%, 41%, and 49%, decrease respectively, *** *p* < 0.001, **** *p* < 0.0001, Figure 7A). The same concentrations of the inhibitor did not cause any effect on MCF7-EV cells (Figure 7A). Similar effects were observed with the inhibitor ZINC00640089 at 0.1 μM, 1 μM, and 10 μM concentration compared with untreated cells (24%, 54%, and 57%, decreases respectively, ** *p* < 0.01, **** *p* < 0.0001, Figure 7B). Figure 7C shows the changes observed in the clonogenic assays after treatment of MCF7, MCF7-EV, and MCF7-LCN2 cells with the inhibitors ZINC00784494 and ZINC00640089. Together, these results suggest that ZINC00784494 and ZINC00640089 inhibit LCN2.

## 3. Discussion

This study found that LCN2 is significantly overexpressed in IBC cells compared to non-IBC cells. LCN2-siRNA silencing reduced colony formation, migration, and invasiveness ability of IBC cells. Moreover, we demonstrated that targeting LCN2-calyx with small molecule inhibitors decreased colony formation and cell viability of IBC cells. Thus, our findings suggest LCN2 as a potential therapeutic target for IBC.

LCN2 is a secreted glycoprotein that can transport iron to different tissues through its association with mammalian siderophores [13,30]. Overexpression of LCN2 has been observed in different types of cancer, such as breast, pancreas, ovarian, thyroid, colon, and bile duct cancers [13,30,31,32]. Moreover, dysregulation of LCN2 at the protein and mRNA level in IBC has been observed and associated with cancer progression [15,16,17]. A recent publication found a significantly higher expression of LCN2 in IBC versus non-IBC tumors, regardless of the molecular subtypes [4]. Our study observed a consistent LCN2 protein overexpression in IBC compared to non-IBC cells, independent of the molecular subtype.

Our work showed that LCN2 silencing achieved a significant decrease in proliferation, migration, and invasion of IBC cells. The observed reduction in the number of colonies upon LCN2 silencing suggests that LCN2 promotes the self-renewal capacity of IBC tumor cells. Similarly, the reduction of the invasion ability of SUM149 following LCN2 silencing suggests a role of LCN2 in the epithelial to mesenchymal transition (EMT) process, a characteristic of the highly metastatic IBC cells [3,33,34]. Reports indicate that a hybrid epithelial/mesenchymal (E/M) phenotype occurs in IBC cells [35,36]. It is speculated that this hybrid E/M phenotype promotes IBC cells clustering together, forming circulating tumor cells (CTCs). CTCs possess a highly metastatic potential and contribute to metastasis [37]. Further studies are needed to assess changes in the expression of epithelial and mesenchymal markers after LCN2 silencing.

We observed cell cycle arrest in the G0/G1 to S phase transition following LCN2 silencing in SUM149 cells. The arrest in cell cycle progression at the G0/G1 to S phase transition was confirmed by increases in the levels of cell cycle inhibitory proteins, p21 and p27, and the decrease in cyclin E1, cyclin E2, and CDK4. Moreover, the changes observed in the apoptotic markers, caspase-3, caspase-9, and PARP-1 indicate that LCN2 silencing activates both cell cycle progression arrest and apoptosis.

Recent efforts to identify specific therapeutic targets for IBC have led to the discovery of several non-specific inhibitors, some in the preclinical stage and others in ongoing clinical trials [3]. For example, the combination of a histone deacetylase inhibitor (HDACi) with nanoparticle albumin-bound paclitaxel is part of the metastatic clinical trial to treat HER2-negative IBC [3,38]. Our study used a structure-based computational approach to identify potential LCN2 inhibitors in the ZINC database of the Asinex library. Based on molecular docking simulations, it was predicted that hit compounds binding to the LCN2-calyx pocket and interfering with key residues Trp79, Arg81, Tyr106, Lys125, and Lys134 would inhibit LCN2 activity and, thus, cell proliferation and viability. Moreover, since there is no evidence of LCN2 inhibitors in the literature, our study generated small molecule inhibitors against LCN2 for the first time.

The inhibitors proposed in this study target the interacting region between LCN2 and its natural ligands, bacterial siderophores, and catecholate [13,26]. As bacterial siderophores are iron carriers, the reduction in cell proliferation and cell viability of IBC cells upon drug treatment could be caused by an impairment in cellular activities due to a shortage in iron uptake. In fact, certain types of cancers can reprogram iron metabolism to allow cancer cells to survive [39]. Although therapies to block iron dependencies have been extensively studied in cancer, there are no therapies to inhibit iron uptake by cancer cells in IBC. Therefore, this study could represent a novel therapeutic approach for IBC.

Molecular docking simulations performed for ZINC00784494 and ZINC00640089 predicted that both compounds would bind to the LCN2-calyx pockets. Clifton and co-workers reported that these pockets are essential for ligand recognition. Therefore, these compounds may potentially inhibit LCN2′s iron transporting ability mediated by its ligands, the siderophores. Trp79, Tyr106, and Lys106 are the possible key residues in the LCN2-calyx that interact with both inhibitors. As reported by Clifton et al., the LCN2 ligand-contacting residues conserved across 18 vertebrate species, including Lys134, Trp79, and Tyr106, which form the three main pockets that define the calyx binding site [26,30,40].

Interestingly, each of the three LCN2 residues interacting with the inhibitors are within this three-pocket assembly. Therefore, the LCN2-inhibitors identified have the potential to block the interaction between LCN2 and its natural ligands in vitro. These predicted interactions may explain the results observed when inhibitor ZINC00784494 and inhibitor ZINC00640089 were tested against non-expressing-LCN2 breast cancer cells (MCF7) and LCN2-overexpressing clones (MCF7-LCN2). Inhibitors ZINC00784494 and ZINC00640089 significantly decreased MCF7-LCN2 cell proliferation, suggesting the specificity of both inhibitors towards the LCN2-calyx. Moreover, the reduction of p-Akt levels after treatment of SUM149 cells with ZINC00784494 and ZINC00640089 inhibitors, further suggests the specificity of these LCN2 inhibitors. Further binding studies are needed to confirm the direct interaction of these inhibitors with LCN2.

As LCN2 plays a pivotal role in cancer, targeting this protein offers a novel opportunity to develop a specific IBC treatment drug. Inhibiting LCN2 using RNAi or small molecule inhibitors offers several advantages over the therapeutic regimens currently available. First, for IBC patients where HER2 or the epidermal growth family receptor (EGFR) are not expressed or mutated, targeting LCN2 is an option. Second, as LCN2 is overexpressed in IBC cells, small inhibitors or RNAi can be combined with chemotherapeutic agents commonly used for IBC treatment. Finally, using nanoliposomal formulations to deliver LCN2-siRNAs, as reported by Guo et al., or using LCN2 inhibitors targeting the LCN2-calyx, as we showed in this study, represent feasible approaches to develop new IBC treatments [32].

## 4. Materials and Methods

### 4.1. Cell Culture

The human IBC cell lines MDA-IBC3 (estrogen receptor and progesterone receptor-negative; HER2 positive), SUM149 (estrogen receptor and progesterone receptor-negative; HER2 negative) were kindly donated by Dr. Bisrat Debeb from the Department of Breast Medical Oncology at MD Anderson Cancer Center, Houston, TX, USA. Cells were cultured in Hams F-12 medium (Thermo Fisher Scientific, Grand Island, NY, USA) supplemented with heat-inactive 10% fetal bovine serum (FBS) (Thermo Scientific, Logan, UT, USA), 0.1% penicillin/streptomycin (Thermo Fisher Scientific, Grand Island, NY, USA), 5 μg/mL insulin from bovine pancreas (Sigma-Aldrich, St. Louis, MO, USA), and 1 μg/mL hydrocortisone (Sigma-Aldrich). Breast cancer cell (BCC) lines MDA-MB-231 (ATCC HTB-26), and SKBR3 (ATCC HTB-30) were purchased from American Type of Culture Collection (ATCC, Chicago, IL, USA) and cultured in Dulbecco’s modified Eagle’s medium (DMEM) supplemented with heat-inactive 10% fetal bovine serum (FBS) (Thermo Fisher Scientific), and 0.1% penicillin/streptomycin (Thermo Fisher Scientific). BCC line MCF7 (ATCC HTB-22D) was purchased from ATCC and cultured in Dulbecco’s Modified Eagle Medium (DMEM) supplemented with heat-inactive 10% FBS (Thermo Fisher Scientific) and 0.1% penicillin/streptomycin (Thermo Fisher Scientific). Cell lines were grown at 37 °C and 5% CO_2_. Experiments were performed at 75% to 85% confluency. Mycoplasma-free cells were always used.

### 4.2. Western Blot Analysis

Cell lysates were collected on ice using lysis buffer (1% Triton X, 150 mM NaCl, 25 mM Tris HCl,0.4 mM NaVO_4_, 0.4 mM NaF and protease inhibitor cocktail from Sigma, St. Louis, MO, USA) and vortexed periodically for 30 min. Lysates were centrifuged for 15 min at 4 °C, and supernatants were collected. Total protein concentrations were determined using Bio-Rad DC Protein Assay reagents (Bio-Rad, Hercules, CA, USA) following the manufacturer’s protocol. Equal amounts of protein for each sample (40 μg to 50 μg per lane) were separated by SDS-PAGE, blotted onto nitrocellulose membranes, blocked with 5% non-fat milk, and probed with the appropriate dilution of the corresponding primary antibody. Once incubated with the primary antibody, membranes were rinsed and incubated with the corresponding HRP-conjugated secondary antibody. Bound antibodies were detected using an enhanced chemiluminescence substrate followed by autoradiography using a FluorChemTM 8900 (Alpha Innotech Corporation, San Leandro, CA, USA). Primary antibodies: anti-LCN2 (AF1757) (24 kDa), (R&D System, Minneapolis, MN, USA); Caspase 9 (9502) (47 kDa), Caspase 3 (9665) (35 kDa), Cleaved Caspase 9 (20750) (37 kDa), Cleaved Caspase 3 (9664) (17/19 kDa), PARP-1 (46D11) (89,116 kDa), CDK4 (D9G3D) (30 kDa), CDK6 (DCS83) (36 kDa), Cyclin E1 (HE12) (48 kDa), Cyclin E2 (4132) (48 kDa), p21/Waf1/Cip1 (12D1) (21 kDa), p27/Kip1 (D69C12) (27 kDa), Akt (4685) (60 kDa), p-Akt (Ser473) (4060) (60 kDa) (Cell Signaling, Danvers, MA, USA); anti-β-actin (42 kDa) (Sigma). Secondary antibodies: anti-goat IgG horseradish peroxidase (HRP) (HAF 109) (R&D Systems, Minneapolis, MN, USA), anti-mouse and anti-rabbit IgG (HRP) (Cell Signaling).

### 4.3. Transient and Stable Transfections

Two different small interference RNA (siRNA) molecules were used to silence human *LCN2* (NC_000009.12). LCN2-siRNA-1: target sequence: 5′-GGAAUGCAAUUCUCAGAGA-3′, LCN2-siRNA-2: target sequence: 5′-CAUGCUAUGGUGUUCUUCA-3′, and a scrambled universal negative control siRNA (NC-siRNA) (SIC001) (Sigma) were transiently transfected at a final concentration of 100 nmol/L. A non-treated (NT) cells (containing transfection reagent, only) were also used. MDA-IBC3 cells (5.5 × 10^4^ cells/mL) or SUM149 (5.0 × 10^4^ cells/mL) were seeded in Petri dishes and twenty-four hours later, siRNAs were mixed with Lipofectamine 2000 RNAiMax transfection reagent (Life Technologies, Carlsbad, CA, USA) at a 1:3 (*v*/*v*) (MDA-IBC3) or 1:1 (*v*/*v*) (SUM149) ratio (siRNA: transfection reagent) in serum and antibiotic-free Opti-MEM medium (Life Technologies, Carlsbad, CA, USA). The transfection mix was incubated for 20 min at room temperature (RT) and then added to the cells. Cells were incubated at 37 °C and collected 24 h (MDA-IBC3 cells) or 48 h (SUM149) after transfection. Transfected cells were used to verify the LCN2 silencing or for in vitro experiments.

Ectopic LCN2 expression was performed in breast cancer MCF7 cells. Human LCN2 open reading frame (LCN2ORF) (RC207685, OriGene, Rockville, MD, USA) or empty vector pCMV6-Entry (MCF7-EV) (PS100001, OriGene, Rockville, MD, USA) was stably transfected into MCF7 cells. MCF7 cells (4.0 × 10^4^ cells/mL) were seeded in 6-well plates and incubated at 37 °C. Twenty-four hours later, 5 μg of LCN2-ORF were mixed with Lipofectamine 2000 RNAiMax transfection reagent (Life Technologies, Carlsbad, CA, USA) at a 1:1 (*v*/*v*) (plasmid: transfection reagent) in serum and antibiotic-free Opti-MEM medium (Life Technologies, Carlsbad, CA, USA) and incubated at 37 °C. Six hours later the medium was replaced with MCF7 culture media and incubated at 37 °C. Forty-eight hours later the antibiotic neomycin was added at a final concentration of 1.8 mg/mL for the selection of transduced MCF7 cells. After 2–3 weeks, independent colonies were picked and cultured separately as independent clones.

### 4.4. Colony Formation Assays

Cell growth was assessed by colony formation assays: MDA-IBC3 cells (5.5 × 10^4^ cells/mL) or SUM149 (5.0 × 10^4^ cells/mL) were seeded into 6-well plates. Twenty-four hours later, siRNAs were added to the cells. Twenty-four hours (MDA-IBC3 cells) or forty-eight hours (SUM149) after transfection, 1500 cells for MDA-IBC3 and 1000 cells for SUM149 were seeded into 10-cm Petri dishes containing Hams F-12 (10% FBS, 0.1% antibiotic/antimycotic solution, 0.001% insulin from bovine pancreas, and 0.005% hydrocortisone), and incubated at 37 °C. Twelve days (SUM149 cells) or 19 days (MDA-IBC3 cells) later, colony-forming cells were stained with 0.5% crystal violet solution. Colonies (with at least 50 cells) were counted under a light microscope (Olympus CKX41) in five random fields with a total magnification of 10×.

### 4.5. Cell Viability Assays

MDA-IBC3 cells (5.5 × 10^4^ cells/mL) or SUM149 (5.0 × 10^4^ cells/mL) were seeded in 96-well plates. Twenty-four hours later, cells were transiently transfected with serial dilutions of LCN2-siRNA and NC-siRNA (12.5 nM, 25 nM, 50 nM, and 100 nM final concentrations) with Lipofectamine 2000 RNAiMax. Seventy-two hours after transfection, the medium was removed, and cell viability was measured using Alamar blue dye (Invitrogen, Carlsbad, CA, USA) as previously described by [41]. Optical density (OD) values were obtained spectrophotometrically in a plate reader (Bio-Rad) after 3 h of dye incubation. In all cases, percentages of cell viability were obtained after blank OD subtraction, taking the values of the untreated cells as a normalization control.

### 4.6. Invasion and Migration Assays

Cell invasion and migration were assessed using the transwell assay. SUM149 (5.0 × 10^4^ cells/mL) were seeded into a 6-well plate and transfected with siRNAs as described for the colony formation assays. Forty-eight hours after transfection, cells were collected and resuspended in serum-free Hams F-12 at 5.0 × 10^4^ cells/mL. Fifty-five μL of Matrigel (Corning, Lowell, MA, USA) was added to the upper part of the transwell chamber of a 24-well plate (BD Biosciences, San Diego, CA, USA; 8-μm pore size) and incubated at 37 °C for 1 h (for invasion assay). Two hundred μL of cells were placed on top of Matrigel of each upper chamber. The lower chamber of the transwell was filled with 200 μL Hams F-12 media (10% FBS) and the plate was incubated at 37 °C for 24 h. The numbers of invaded or migrated cells were calculated as previously described [5].

### 4.7. In Vitro Testing of Small Molecules Inhibitors

For cell viability, SUM149 (5.0 × 10^4^ cells/mL) were seeded in 96-well plates. Twenty-four hours later, cells were treated with serial dilutions (0.01 µM, 0.1 µM, 1.0 µM, 10 µM, and 100 µM, final concentrations) of the LCN2 inhibitors. Seventy-two hours after treatment, cell viability was performed as above described, taking the DMSO (1%) treated OD values as the normalization control. For colony formation assays, SUM149 cells (5.0 × 10^4^ cells/mL), MCF7 (4.5 × 10^4^ cells/mL), MCF7-LCN2 (4.5 × 10^4^ cells/mL), or MCF7-EV (4.5 × 104 cells/mL) were seeded into 24-well plates and incubated at 37 °C. Twenty-four hours later, LCN2 inhibitors bought to Asinex corporation (North chestnut, NC, USA) were dissolved in DMSO (0.2%) and added to the cells at 10 µM, 1 µM, and 0.1 µM (final concentrations). NT cells and DMSO (0.2%) treated cells were used as controls. SUM149 (500 cells) and MCF7 (3000 cells) cells were seeded in 6-well plates per treatment for colony formation assays as above described.

### 4.8. Akt/p-Akt Measurements

SUM149 cells (5.0 × 10^4^ cells/mL) were seeded in 10-cm Petri plates and incubated at 37 °C. Twenty-four hours later, cells were treated with the LCN2 inhibitors ZINC00784494 and ZINC00640089 at 10 µM and 1 µM concentrations. NT and DMSO (0.2% final concentration) treated cells were used as controls. Cell pellets of each condition were collected at 15 min, 1 h, and 24 h after drug treatment. The p-AKT Akt and Akt protein levels were assessed by western blots as above described.

### 4.9. Caspase-3 Fluorometric Assay

Caspase-3 activity was assessed using a caspase-3/CPP32 fluorometric assay kit (BioVision, CA, USA) as described in the manufacturer’s protocol, with some modifications. Briefly, SUM149 (5.0 × 10^4^ cells/mL) and MDA-IBC3 (5.5 × 10^4^ cells/mL) were seeded in 10 cm Petri dishes and transiently transfected with siRNAs as above described. Docetaxel (0.5 nM final concentration) was used as a positive control. Seventy-two hours post-transfection cells were collected, protein extracts were obtained and incubated with the Asp-Glu-Val-Asp (DEVD) peptide substrate—which is conjugated to the 7-amino-4-trifluoromethyl coumarin (AFC)—at 37 °C for 60 min. Releasing of AFC was measured with a fluorometric plate reader (Varioskan LUX, Thermo Fisher Scientific, Waltham, MA, USA) at an excitation wavelength of 400 nm and an emission wavelength of 505 nm. The fold-change increase in caspase-3 activity was determined by comparing the release of AFC from the siRNA-transfected cells with the AFC release by the untreated cells.

### 4.10. Flow Cytometry Analysis

To assess cell cycle progression, SUM149 cells were transfected with siRNAs as described above. Forty-eight and seventy-two hours later, attached cells were collected, washed in ice-cold PBS, fixed with 70% cold ethanol, and stored at 4 °C. Twenty-four hours later, cells were washed with ice-cold PBS, resuspended in propidium iodide (PI)/RNase Staining Buffer (BD Biosciences), incubated in the dark for 15 min at room temperature, and then analyzed by flow cytometry in FACS Calibur (BD Biosciences). FLOWJO Software (BD Biosciences) was used to determine the percentage of cells in each phase of the cell cycle.

### 4.11. Molecular Docking

We used PyRx virtual screening tool version 0.8 which uses AutoDock Vina and AutoDock 4 as a docking software with the Lamarckian genetic algorithm as the scoring function for higher docking accuracy, AutoDockTools to generate input files, and Phyton as a programming/scripting language (29). The target protein Lipocalin 2 (crystal structure of the macromolecule, X-ray diffraction 2.19 Å) was retrieved from the Protein Data Bank website (https://www.rcsb.org/, accessed on 15 August 2019) with PDB ID: 3HWG as a PDB file format and prepared using AutoDock 4 by eliminating water molecules and the bound ligands from the calyx binding site and saved as PDBQT file format. Asinex screening library of 25,000 ligands was used to retrieve 2D structures in SDF file format, and further using Open Babel software, these compounds were converted to PDB file format. The grid box coordinates (Vina Search Space) were located at the center of Lys134 with dimensions (Å) of X: 30.0925, Y: 760246, Z: 60.8653 to dock all the ligands where 8 maximum exhaustiveness was calculated for each ligand. Lys134 is a key residue located at pocket #2 that has been identified to form hydrogen bonding interactions with the siderophores of LCN2. All other parameters of software were kept as default, and all bonds contained in ligands were allowed to rotate freely and considering macromolecule as rigid. The results from the PyRx tool and virtual screening are obtained in the CSV or SDF file format for further analysis and data organization. The final visualization of the docked structure was performed using PyMOL v2.4 (www.pymol.org, accessed on 15 August 2019) and was used for protein alignment and as a molecular viewer to generate high-quality molecular structures.

### 4.12. Physicochemical and Pharmacokinetic Properties

Physicochemical and pharmacokinetic properties including absorption, distribution, metabolism (ADME), lipophilicity, water-solubility, drug-likeness, and the PAINS model, were predicted using the SwissADME server [6]. The physicochemical properties values are computed using OpenBabel v2.3.0. The lipophilicity is calculated using five predicted models including XLOGP3, WLOGP, MLOGP, SILICOS-IT, and iLOGP. The water solubility is calculated by the server using the ESOL model and a modified version of the general solubility equation (GSE) model. The pharmacokinetic properties adapt the support vector machine (SVM) algorithm to estimate substrate for the P-gp or inhibitor for the most important CYP isoenzymes. The Lipinski rule of five filters used for drug-likeness prediction is implemented from reference [42]. The PAINS model implements a rule-based method for lead-likeness, which was adapted from reference [43].

### 4.13. Statistical Analysis

All experiments were performed at least in triplicate. Graphs were constructed with the GRAPH PAD Prism 8 software (GraphPad Software, Inc., La Jolla, CA, USA). Data were analyzed using Student’s *t*-test for comparing two groups and ANOVA tests for multiple group comparisons, with *p* < 0.05 considered statistically significant (* *p* < 0.05, ** *p* <0.01, *** *p* < 0.001).

## 5. Conclusions

Our study provides evidence that LCN2 is abundant in IBC cells and that LCN2 silencing decreased cell proliferation, cell migration, and reduced the invasiveness ability of IBC cells. In vitro targeting of LCN2 with small molecule inhibitors suggests the potential of LCN2 as a plausible target for IBC treatment. Together, these findings open the possibility of a novel targeted therapeutic approach for an aggressive and deadly invasive cancer, such as IBC.

## 6. Patents

An invention report related to this publication has been submitted to the NIH through the Iedison.Gov database, with a unique Extramural Invention Report Number of 0578705-19-0003 (Docket number 19-0003).

## Data Availability

The data presented in this study are available on request from the corresponding author.

## Supplementary Materials

The following are available online at https://www.mdpi.com/article/10.3390/ijms22168581/s1.
