# Supplementary material for: Targeting Lipocalin-2 in Inflammatory Breast Cancer Cells with Small Interference RNA and Small Molecule Inhibitors
Source: Int J Mol Sci. 2021 Aug 10;22(16):8581. doi: 10.3390/ijms22168581 (PMC8395282; doi:10.3390/ijms22168581)
Supplement: Supplementary file 1 [file ijms-22-08581-s001.zip › ijms-1285824-supplementary.pdf]

Targeting Lipocalin-2 in Inflammatory Breast Cancer Cells with Small Interference RNA and Small Molecule Inhibitors

Ginette S. Santiago-Sánchez, Ricardo Noriega-Rivera, Eliud Hernández-O'Farrill, Fatma Valiyeva, Blanca Quiñones-Diaz, Emilly S. Villodre, Bisrat G. Debeb, Andrea Rosado-Albacarys, and Pablo E. Vivas-Mejía

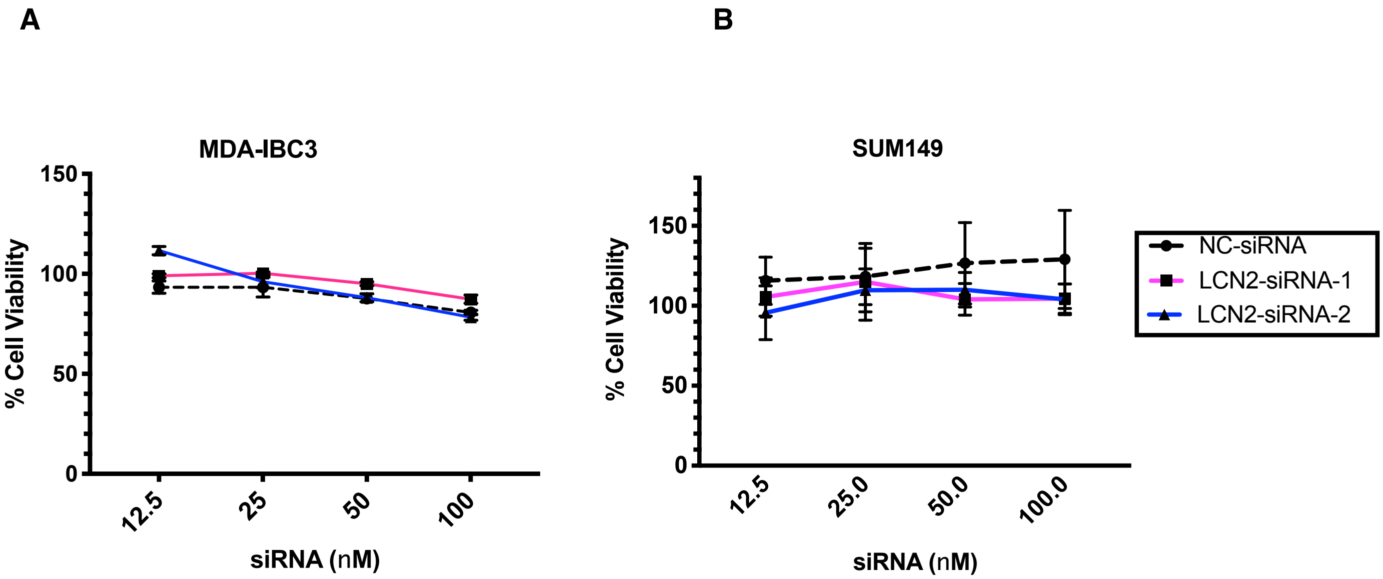

Figure S1| Effect of LCN2 silencing on cell viability of IBC cells. Cell viability was assessed in (A) MDA-IBC3 and (B) SUM149 with Alamar Blue dye 72 hours after negative control (NC-siRNA), LCN2-siRNA-1, and LCN2-siRNA-2 transfection. No significant difference was observed between LCN2-siRNAs and NC-siRNA. Results are shown as Mean ± SEM of triplicate experiments.

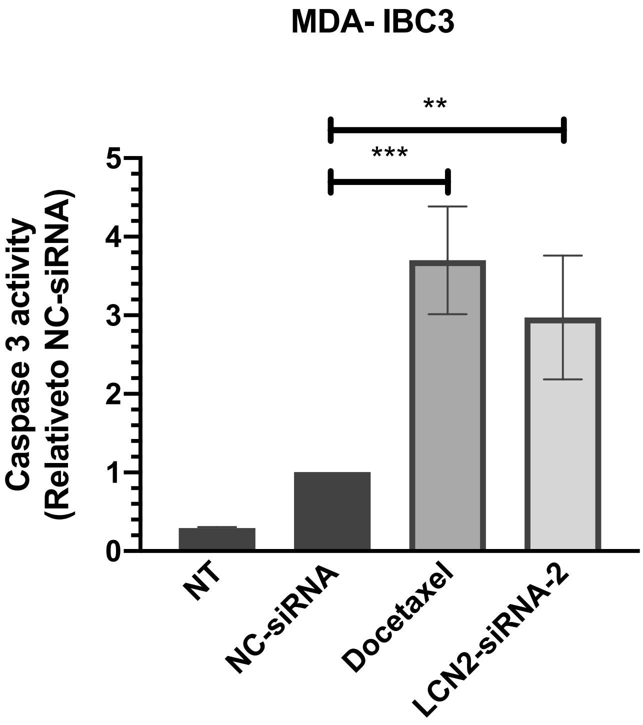

Figure S2 | LCN2-siRNA-based silencing induces Caspase-3 activation in MDA-IBC3 cells. Caspase-3 fluorometric activity assay in MDA-IBC3 cells after LCN2-siRNA-2 and NC-siRNA transfection, and docetaxel treatment as positive control. Results are shown as Mean ± SEM of triplicate experiments (**P<0.01, ***P<0.001).

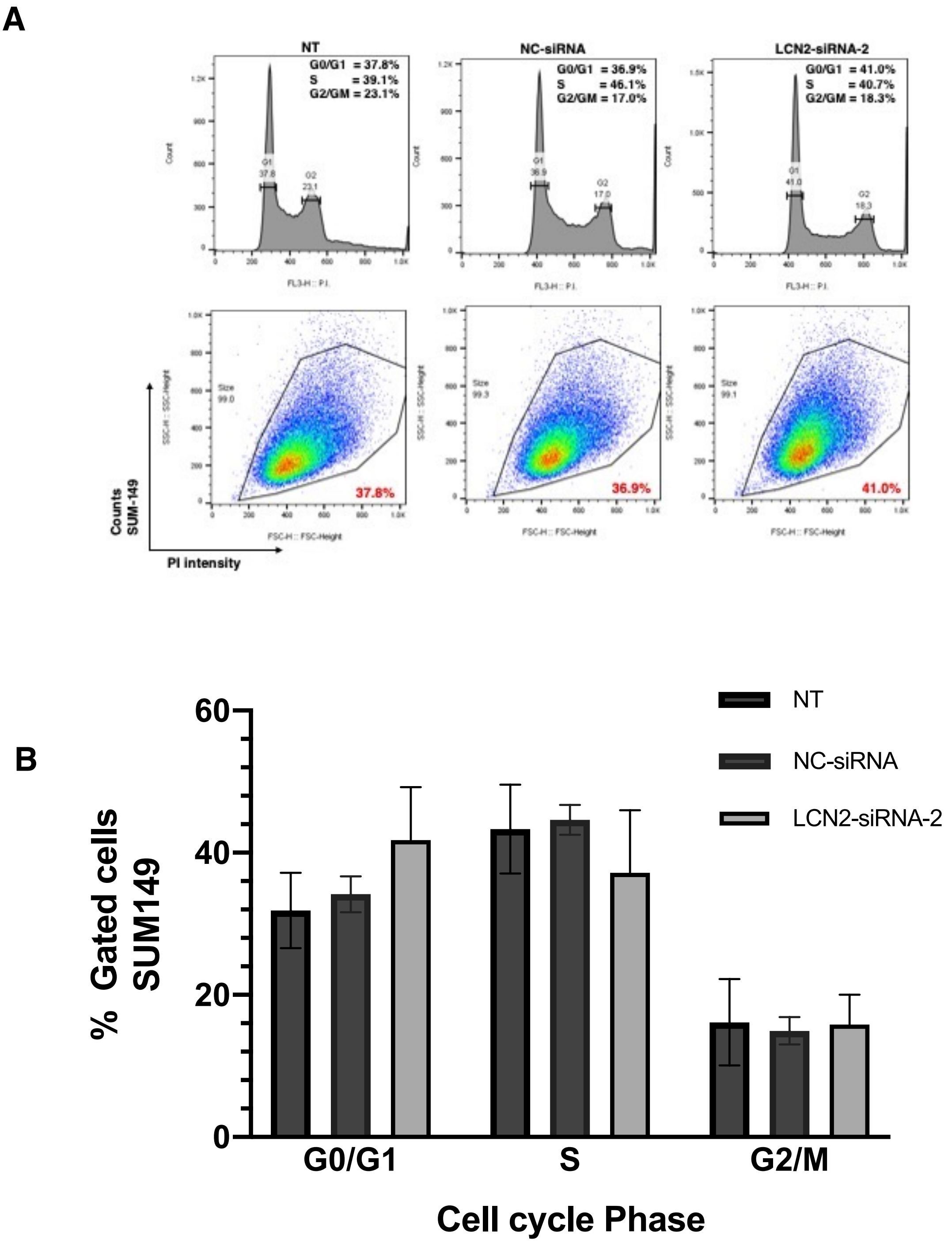

Figure S3| Assessment of cell cycle progression was performed by flow cytometry after LCN2-siRNA silencing in IBC cells. (A) Histogram showing a tendency in cell cycle arrest at G0/G1 to S phase transition 48 hours after LCN2-siRNA-2 transfection in SUM149 cells compared to NC-siRNA. (B) Quantification of flow cytometry data showing an increase in SUM-149-LCN2-siRNA-2 (41.8%) transfected cells at G0/G1 to S phase transition compared to NC-siRNA (34.2%).

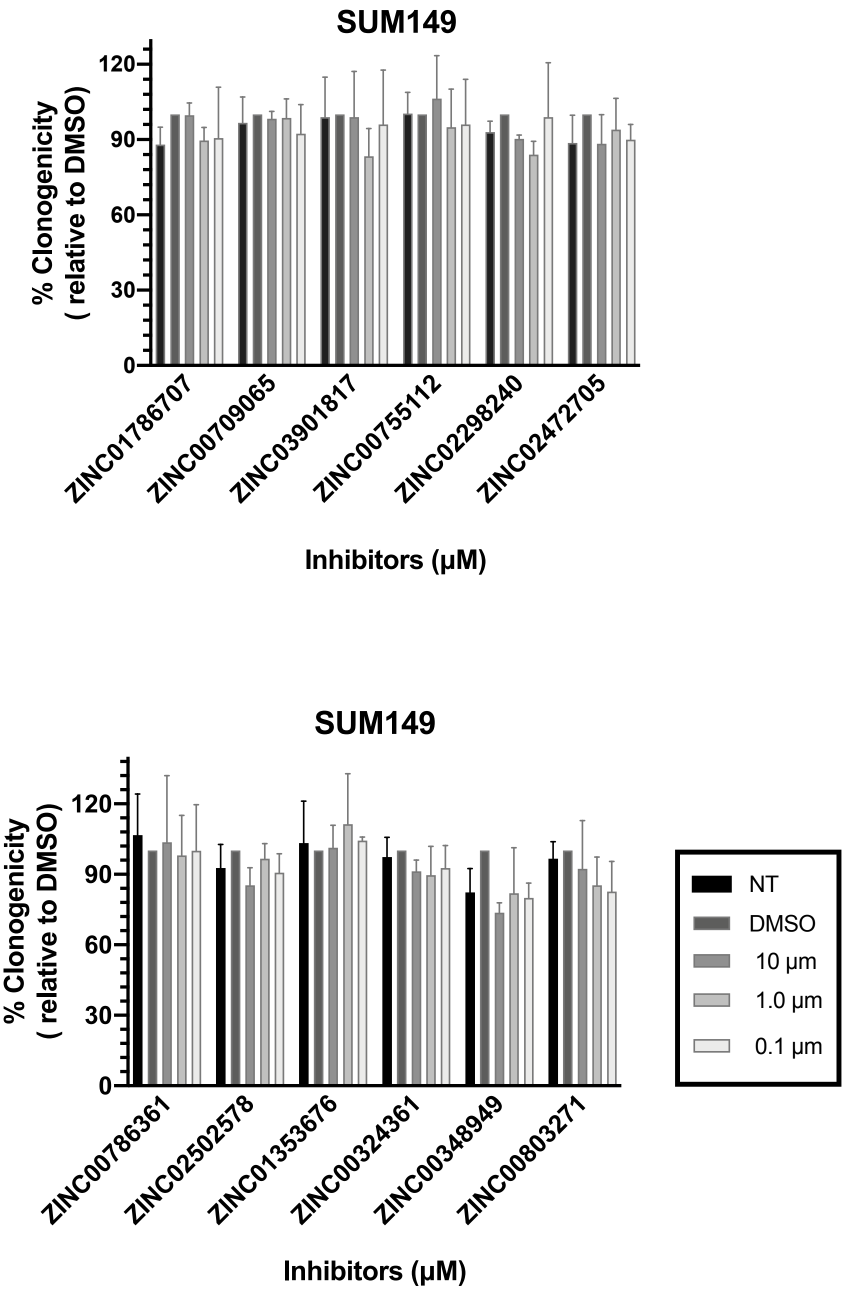

Figure S4| LCN2 inhibitors not showing reduction in cell proliferation in IBC cells. Colony formation assay was performed in SUM-149 cells after treatment with LCN2 inhibitors at 10 μM, 1 μM, and 0.1 μM. The percentage of clonogenicity was calculated relative to DMSO. Results are shown as Mean ± SEM of triplicate experiments.

Table S1 l Receptor expression in IBC and non-IBC cell lines.

Inflammatory Breast Cancer Cell Lines: IBC-3 (PR-, ER-, HER2+), SUM-149 (PR-, ER-, HER2-). Non-Inflammatory Breast Cancer Cell Lines: metastatic (MDA-MB-435 (ER-, PR, HER2+), MDA-MB-231 (ER-, PR-, HER2-), and non-metastatic (MCF7 (ER+, PR+, HER2-).

Breast Cancer Lines	Markers			
IBC	ER	PR	HER2	
SUM-149	–	–	–	Metastatic
MDA-IBC3	–	–	+	Metastatic
NON-IBC				
SKBR-3	–	–	+	Metastatic
MDA-MB-231	–	–	–	Metastatic
MDA-MB-435	–	–	+	Metastatic
MCF7	+	+	–	Non-Metastatic

Table S2: Predicted physicochemical, lipophilicity, water solubility, druglikeness, and PAINS properties of LCN2 inhibitors and compound derivatives. [a] MW = molecular weight (g/mol). [b] TPSA = Topological polar surface area (Å^2). [c] Lipinski rule-of-five: number of violations. [d] PAINS: Pan Assay Interference Structures alert identify potentially problematic fragments.

Molecule ID	Physicochemical Properties					Lipophilicity (LogP)	Water solubility		Druglikeness		PAINS[d]
	MW[a]	TPSA[b]	Rotatable bonds	H-bond acceptors	H-bond donors		Log*S*	Solubility	Lipinski[c]	Bioavailability score	
ZINC00829534	368.43	50.5	3	3	0	3.81	-5.08	Moderately soluble	0	0.55	0
ZINC02472705	312.32	58.89	1	3	1	3.97	-5.19	Moderately soluble	0	0.55	0
ZINC02298240	313.31	56.24	1	4	0	4.15	-5.37	Moderately soluble	0	0.55	0
ZINC00348949	312.32	47.51	1	3	0	3.72	-5.36	Moderately soluble	0	0.55	0
ZINC03901817	353.33	71.52	1	4	0	3.03	-4.57	Moderately soluble	0	0.55	1
**ZINC00640089**	370.32	51.1	5	5	1	4.08	-4.86	Moderately soluble	0	0.55	0
ZINC02502578	338.36	51.96	3	3	0	3.83	-4.87	Moderately soluble	0	0.55	0
ZINC00230567	332.36	83.98	7	4	2	2.35	-4.23	Moderately soluble	0	0.55	0
ZINC00324361	329.35	47.89	3	4	0	4.17	-5.17	Moderately soluble	0	0.55	1
ZINC00709065	357.36	76.38	4	4	1	3.61	-4.79	Moderately soluble	0	0.55	0
**ZINC00784494**	362.4	100.44	4	4	1	3.83	-5.05	Moderately soluble	0	0.55	0
ZINC00755112	369.42	65.08	1	4	0	3.82	-5.44	Moderately soluble	1	0.55	0
ZINC00786361	394.47	58.2	8	2	2	4.4	-5.66	Moderately soluble	1	0.55	0
ZINC00803271	350.8	51.1	4	2	1	3.92	-4.9	Moderately soluble	0	0.55	0
ZINC01353676	369.42	89.21	5	5	0	3.24	-4.63	Moderately soluble	0	0.55	0
ZINC01786707	291.26	64.96	1	5	0	2.84	-3.77	Soluble	0	0.85	1

Table S3: Predicted pharmacokinetic properties of LCN2 inhibitors and compound derivatives.[a] Pgp: P glycoprotein substrate. [b] Log K_p (cm/s): skin permeation

Molecule ID	Pharmacokinetic Properties								
	GI absorption	BBB permeant	Pgp[a] substrate	CYP1A2 inhibitor	CYP2C19 inhibitor	CYP2C9 inhibitor	CYP2D6 inhibitor	CYP3A4 inhibitor	log Kp (cm/s)[b]
ZINC00829534	High	Yes	Yes	Yes	Yes	Yes	No	Yes	-5.62
ZINC02472705	High	Yes	No	Yes	Yes	No	Yes	No	-5.08
ZINC02298240	High	Yes	No	Yes	Yes	No	No	No	-4.9
ZINC00348949	High	Yes	No	Yes	Yes	No	No	No	-4.89
ZINC03901817	High	Yes	No	Yes	Yes	Yes	No	Yes	-6.08
**ZINC00640089**	High	Yes	No	Yes	Yes	Yes	Yes	No	-5.68
ZINC02502578	High	Yes	No	Yes	Yes	Yes	No	No	-5.54
ZINC00230567	High	No	No	No	Yes	Yes	Yes	Yes	-5.78
ZINC00324361	High	Yes	No	Yes	Yes	Yes	No	No	-4.92
ZINC00709065	High	No	No	Yes	Yes	Yes	No	No	-5.72
**ZINC00784494**	High	No	No	Yes	Yes	Yes	No	Yes	-5.55
ZINC00755112	High	Yes	Yes	Yes	Yes	Yes	No	No	-5.38
ZINC00786361	High	Yes	Yes	No	Yes	Yes	Yes	Yes	-4.91
ZINC00803271	High	Yes	No	Yes	Yes	Yes	No	Yes	-5.49
ZINC01353676	High	No	No	Yes	Yes	Yes	No	No	-5.91
ZINC01786707	High	Yes	No	Yes	Yes	Yes	No	No	-6.06

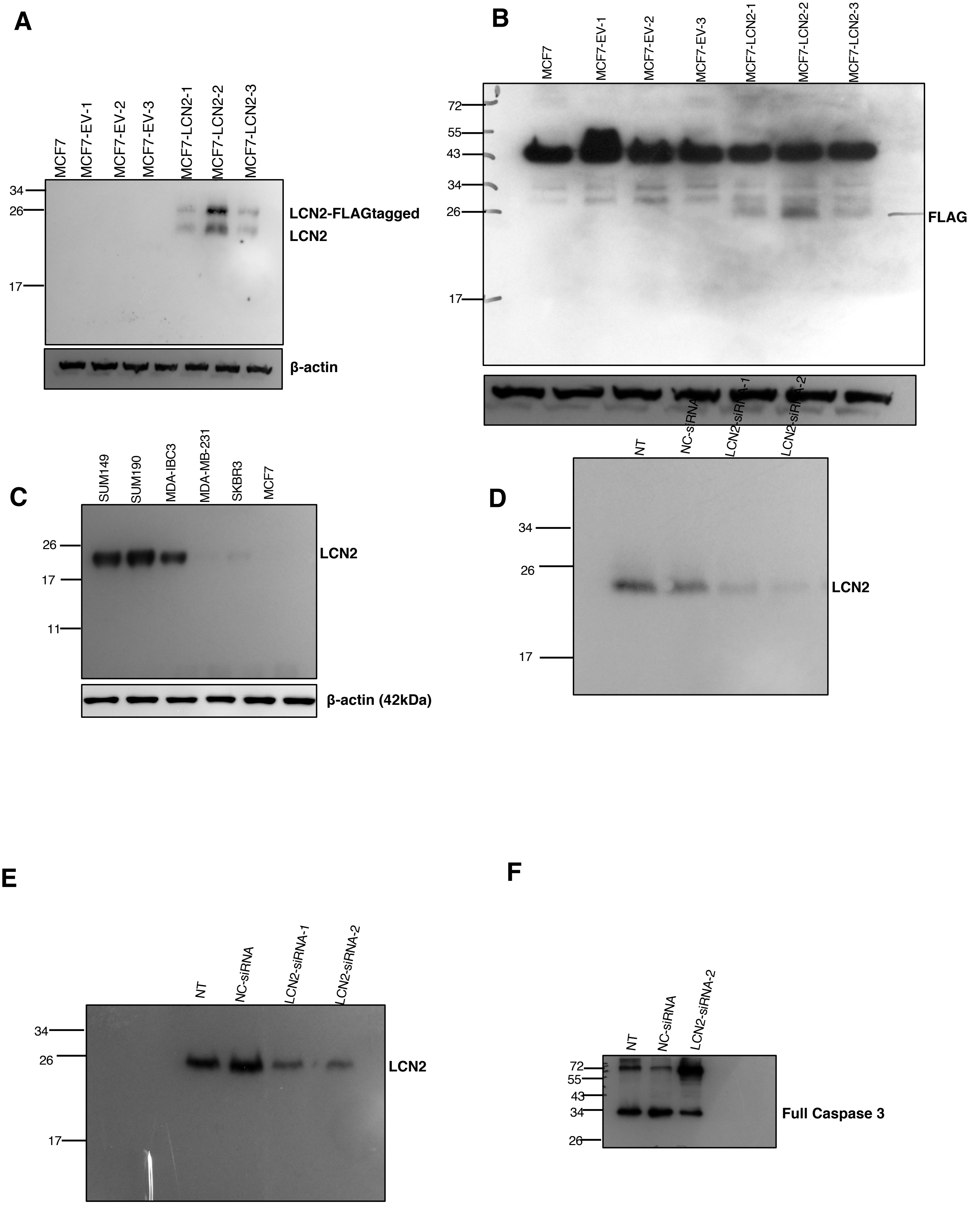
A
MCF7
MCF7-EV-1
MCF7-EV-2
MCF7-EV-3
MCF7-LCN2-1
MCF7-LCN2-2
MCF7-LCN2-3
34
26
17
LCN2-FLAGtagged
LCN2
β-actin
B
MCF7
MCF7-EV-1
MCF7-EV-2
MCF7-EV-3
MCF7-LCN2-1
MCF7-LCN2-2
MCF7-LCN2-3
72
55
43
34
26
17
FLAG
C
SUM149
SUM190
MDA-IBC3
MDA-MB-231
SKBR3
MCF7
26
17
11
LCN2
β-actin (42kDa)
D
NT
NC-siRNA
LCN2-siRNA-1
LCN2-siRNA-2
34
26
17
LCN2
E
NT
NC-siRNA
LCN2-siRNA-1
LCN2-siRNA-2
34
26
17
LCN2
F
NT
NC-siRNA
LCN2-siRNA-2
72
55
43
34
26
Full Caspase 3

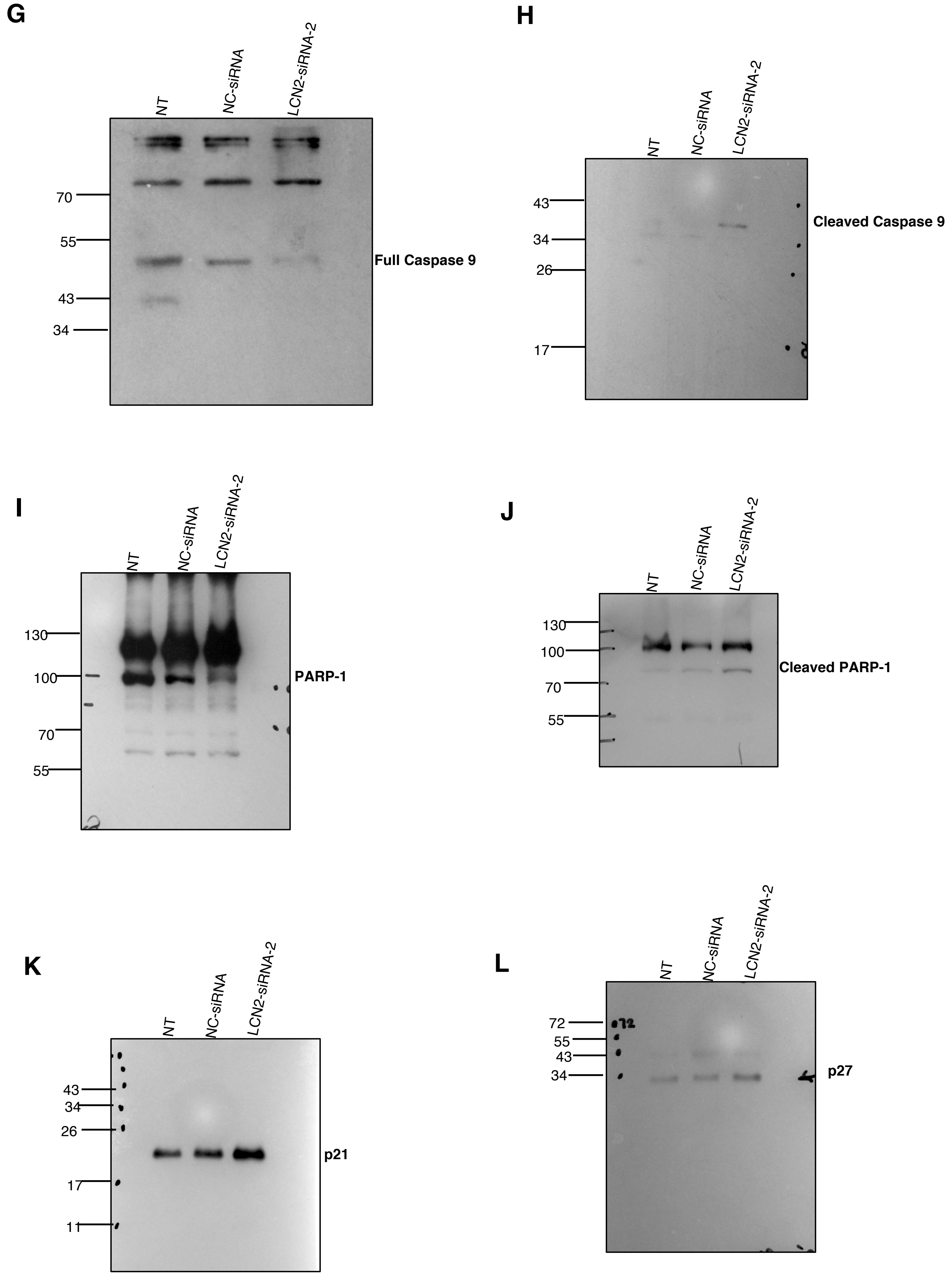
G
NT
NC-siRNA
LCN2-siRNA-2
70
55
43
34
Full Caspase 9
H
NT
NC-siRNA
LCN2-siRNA-2
43
34
26
17
Cleaved Caspase 9
I
NT
NC-siRNA
LCN2-siRNA-2
130
100
70
55
PARP-1
J
NT
NC-siRNA
LCN2-siRNA-2
130
100
70
55
Cleaved PARP-1
K
NT
NC-siRNA
LCN2-siRNA-2
43
34
26
17
11
p21
L
NT
NC-siRNA
LCN2-siRNA-2
72
55
43
34
p27

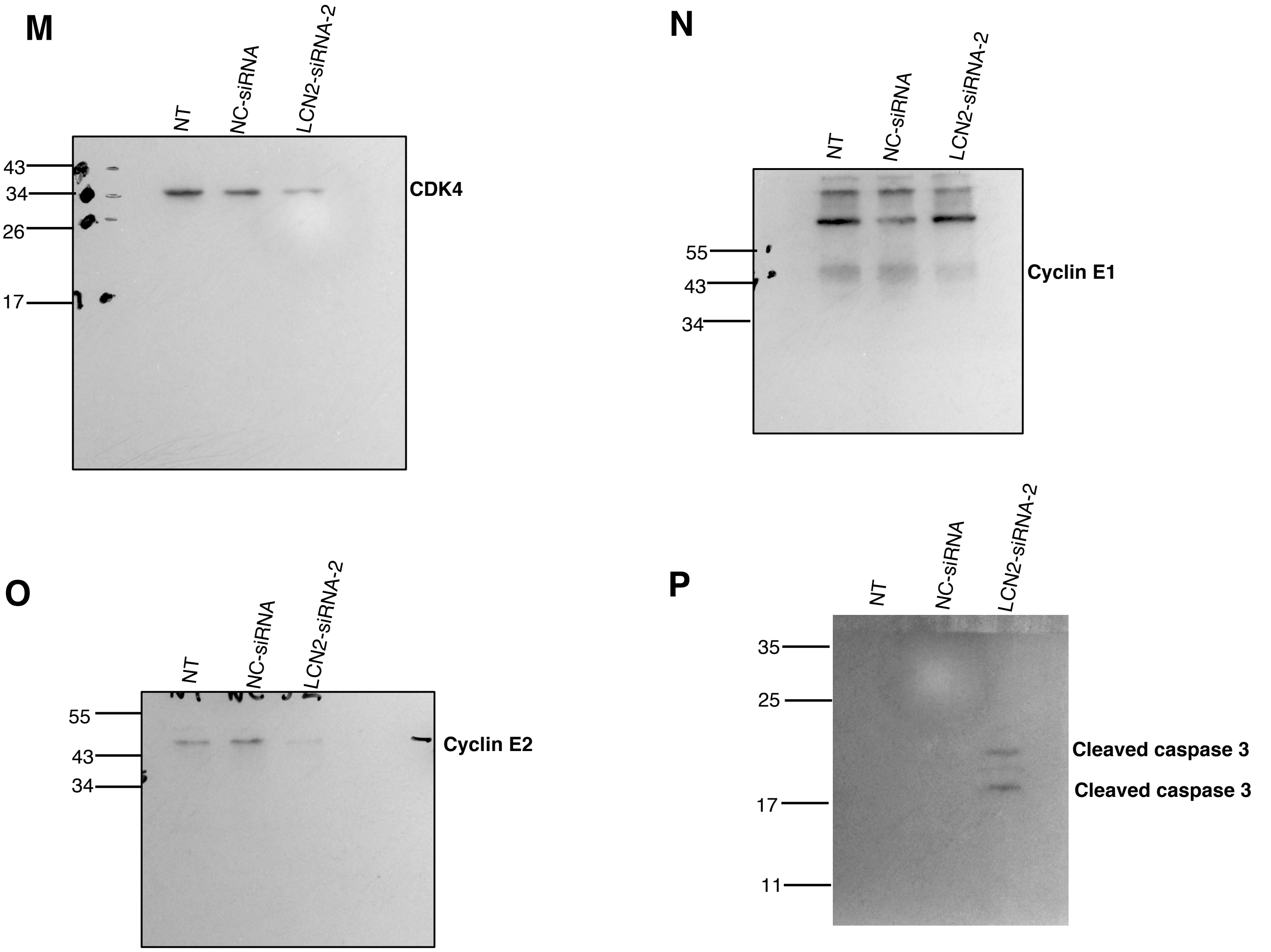

Figure S5| Western blot images. Protein bands and molecular weight markers are shown for (A) LCN2 (B) LCN2-FLAG-tagged, (C) Figure 1A, (D) Figure 1C, (E) Figure 1D, (F) Figure 2B, (G) Figure 2B, (H) Figure 2B, (I) Figure 2B, (J) Figure 2B, (K) Figure 2E, (L)) Figure 2E, (M)) Figure 2E, (N)) Figure 2E, (O) Figure 2E, and (P) Figure 3B.
